# Possible use of 2D shear wave liver elastography in new-onset ascites evaluation

**DOI:** 10.1186/s12876-024-03159-1

**Published:** 2024-02-08

**Authors:** Andrej Hari, Borut Štabuc

**Affiliations:** 1 Department of gastroenterology and hepatology, University Medical Centre Ljubljana, Ljubljana, Slovenia; 2 Department of gastroenterology and hepatology, University Medical Centre Ljubljana, Ljubljana, Slovenia

**Keywords:** Ultrasound, Quality criteria, Cirrhosis, Carcinomatosis, Liver

## Abstract

**Background:**

No data on the use of 2D shear wave elastography exists regarding the evaluation of the new-onset ascites causality.

**Aims:**

To determine whether 2D shear wave elastography can help in the non-invasive assessment of the new-onset ascites cause. To assess the applicability of liver stiffness measured by 2D shear wave elastography using Esaote MyLab Nine apparatus in patients with ascites.

**Methods:**

In 52 consecutive patients with new-onset ascites (January 2020 to October 2021), liver stiffness using 2D shear wave elastography was prospectively measured. The reliable measurements were used for further analysis. Relevant clinical and laboratory data was collected.

**Results:**

The calculated liver stiffness measurement cut-off value of 14.4 kPa held 94% accuracy, 100% sensitivity, and 83% specificity when determining ascites with serum ascites albumin gradient ≥11 g/L. Reliable 2D shear wave elastography success rate was 84%.

**Conclusions:**

2D shear wave elastography may potentially be used to differentiate transudative from exudative ascites, especially in patients with portal hypertension and peritoneal carcinomatosis.

## Introduction

Ascites means the appearance of free fluid in the abdominal cavity. The most common processes involved in its occurrence are portal hypertension (PH) and peritoneal membrane disease. In the Western population, the most common cause of ascites is PH, which is due to cirrhosis in most cases [1–3]. The clinically relevant ascites division is based on the serum-ascites albumin gradient (SAAG) determination, which biochemically divides ascites into exudate and transudate. While albumin-rich ascites (exudate) is due to inflammatory and/or neoplastic causes, ascites due to PH is a transudate characterized by SAAG values ≥ 11 g/L; this value has more than 97% accuracy in differentiating portal hypertensive causes from other causes [1–3]. However, several etiologies may be present simultaneously (mixed ascites), which complicates differential diagnosis [2, 4–6]. The most common causes of ascites are portal hypertension, neoplastic causes and inflammatory causes, or pancreatic ascites.

In particular, it is clinically very important to rapidly classify ascites into benign (mostly due to PH) and malignant (due to peritoneal carcinomatosis); more than 90% of new-onset ascites cases belong to one of these two etiologies [2, 6, 7]. In the presence of ascites, findings typical of cirrhosis such as morphological changes and PH-specific signs such as the presence of portosystemic collaterals (Table 2) can be used to postulate PH as the etiology [8, 9]. Imaging signs of cirrhosis and PH have high inclusion and low exclusion value.

Liver elastography is commonly used to further improve the cirrhosis and PH diagnosis [8, 9]. According to the Baveno VII consensus conference [10], the presence of clinically significant portal hypertension (CSPH) in patients with compensated advanced chronic liver disease (cACLD) can be reliably ruled out by liver elastography when the elastographic value is < 15 kPa and platelet count > 150 × 10^9^ per nanoliter of blood. The confirmatory threshold of elastography for CSPH in the most common cACLD etiologies is > 25 kPa, and from 15 kPa onward, the probability of CSPH presence slowly increases [10]. 2D shear wave elastography (2D-SWE) is a well validated method in this field [11–13]. In two studies performed in a subset of patients with cirrhosis with or without ascites, 2D-SWE showed a significantly positive correlation with invasively evaluated portal pressure [14, 15].

A study by Kolhaas et al. on a phantom model and using TE (XL probe) found that, in cases where the thickness of the perihepatic ascites layer did not exceed 20 mm, a non-hepatic cause of ascites could be reliably identified [16]. A clinical study performed by Bota et al. identified a good predictive value of point shear elastography (pSWE) to distinguish cirrhotic and non-cirrhotic etiology of ascites [17].

Given the wide availability of elastographic methods, we are interested in whether 2D-SWE can provide data allowing a non-invasive discrimination between PH-related causes and other causes in patients with new onset ascites. There is no study data in this area. Also, the applicability of 2D-SWE measurement using Esaote MyLab Nine apparatus in patients with ascites will be concurrently assessed.

## Materials and methods

We obtained the approval of the regional General Hospital Celje Ethics Commission and performed a retrospective analysis of the prospectively collected data. Informed written consent was obtained from each patient included in the study. The study protocol conformed to the ethical guidelines of the Declaration of Helsinki and Istanbul.

### Study population

Data collection, blood and ascites analysis, and ultrasound examination were performed between January 2020 and October 2021. Based on similarly designed studies, the size of our hospital center, and the statistical characteristics of the observed parameters, we conducted a study on a sample of 50 patients. The cohort was divided according to the SAAG value into Group 1 (SAAG ≥11 g/L) and Group 2 (SAAG < 11 g/L). Based on a preliminary statistical analysis, we decided to include at least 30 patients in Group 1 and 15 patients in Group 2. The cohort was completed when both conditions were met.

Our study included patients with new-onset ascites who were hospitalized in the internal medicine wards of the General Hospital Celje. The inclusion criteria were as follows: ultrasonographic confirmation of clinically relevant ascites (grade 2 or 3) and hemodynamically stable patients who did not require acute dialysis or vasopressor therapy. No previously known medical condition affecting the onset of ascites. Furthermore, for Group 1, there were as follows: the presence of liver cirrhosis (US or CT morphological signs, laboratory signs of impaired liver function according to the CHILD score) and only patients with sinusoidal PH. The exclusion criteria were as follows: extrahepatic cholestasis presence, portal venous thrombosis presence or thrombosis in any of the hepatic veins, the presence of cardiac cirrhosis, clinical signs of congestive right-sided heart failure, ultrasound-proven dilatation of the right hepatic vein > 10 mm at the characteristic site, the presence of cancer or infiltrative liver disease at the site of elastographic measurement, the presence of a mixed cause of ascites, serum bilirubin > 100 mmol/L and aminotransferase levels (AST, ALT) > 3 times the normal upper limit, and ascites due to bleeding or hollow organ perforation. Furthermore, for Group 1: the presence of spontaneous bacterial peritonitis, acute-on-chronic liver failure (ACLF), active varicose hemorrhage or hepatic encephalopathy Grade > 2.

Sex, age, standard laboratory data on ascites and liver tests, mortality of patients, and data regarding the etiology of ascites were collected using our center’s electronic case record system.

### Blood and ascites analysis

Probatory ascites puncture and peripheral venous blood collection were performed on each patient on the same day. Ascites puncture was performed in all cases with an ultrasound-guided selection of the puncture site. Serum and ascites albumin values ​​were obtained from concomitantly collected samples. The SAAG calculation was performed from the defined values. The performed biochemical analysis of ascites included standard parameters (albumin, leukocytes, and neutrophilic granulocytes values, amylase, creatinine, urea, glucose, etc.). At least 20 ml of ascites was used for a cytological cellular analysis. The analysis was repeated up to 3 times in case of unclear results.

### 2D-SWE and ultrasound examination

**Fig. 1:**
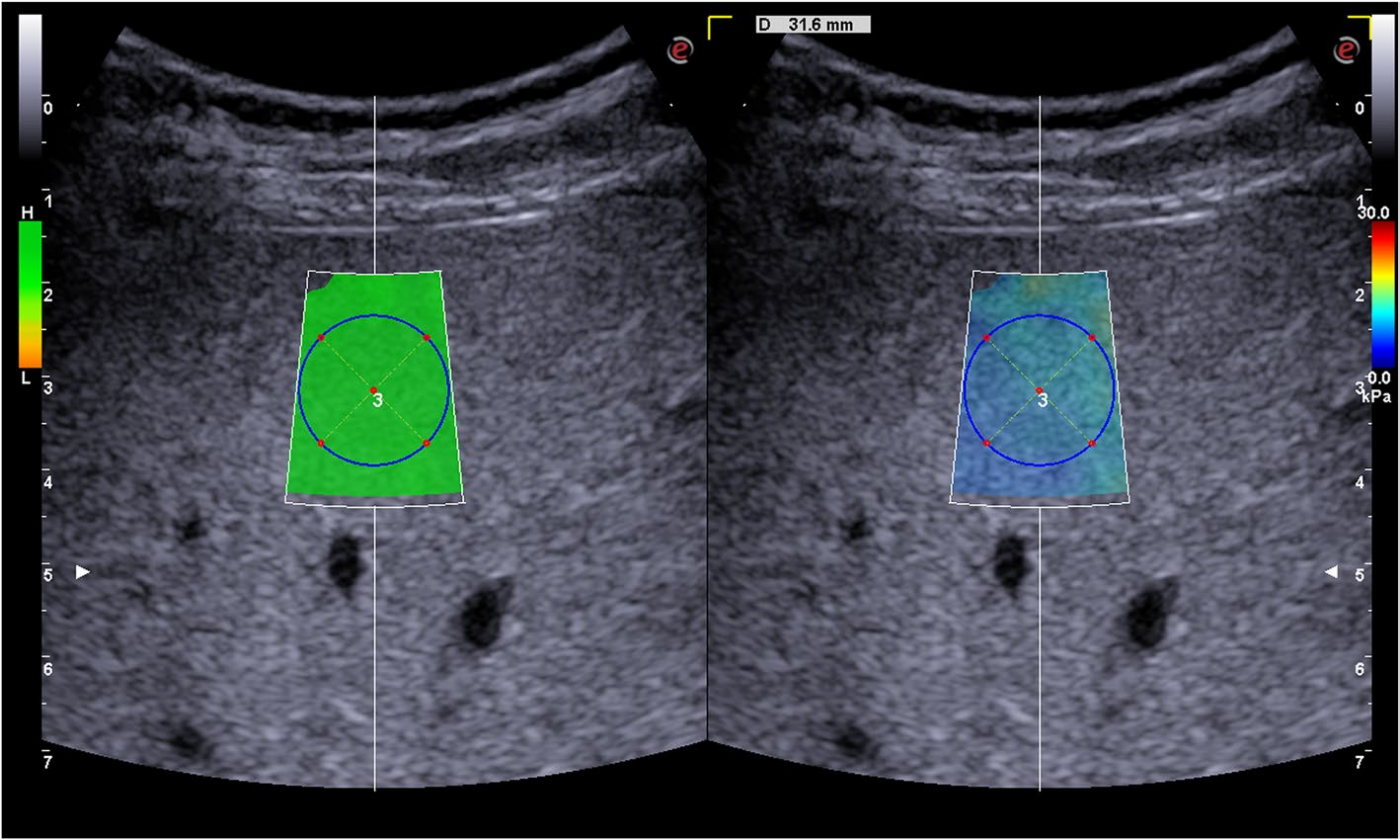
Shear wave measurement example. Left screen: orange to green colour scale – low to high measurement’s quality according to the manufacturer. Right screen: blue to red colour scale – low to high liver stiffness

Each patient underwent 2D-SWE of the liver and an assessment of morphological cirrhosis signs by an investigator with > 5 years of experience. The investigator was not aware of the ascites etiology. The subject fasted for at least 6 h and lay on their back during the examination. The 2D-SWE measurement was obtained during a deep exhalation or in a neutral breathing position. All measurements were performed using the Esaote MyLab Nine™ (Genoa, Italy) device with a monocrystalline high-frequency abdominal probe while following the manufacturer’s protocol for successful measurement. A one-shot image capture function was used during each measurement (Fig. 1). The investigation was conducted in the ultrasound department of the Department of Gastroenterology, General Hospital Celje.

Elastography was performed with the lowest possible perihepatic amount of ascites, and paracentesis was performed before the examination if necessary (Table 1). Reliable elastographic measurement study protocol for Esaote MyLab Nine was defined according to established recommendations, study data [18–24], and manufacturer’s recommendations (Table 1).

**Table 1:** Reliable liver stiffness measurement

Reliable liver stiffness measurement using Esaote 2D-SWE	
6 successful measurements	
> 60% success of all the measurements taken	
IQR/LSM < 30%	
ROI > 10 mm in all measurements	
SD <3x of LSM in all measurements	
Depth of the measurement < 50 mm	
Measurement > 1 cm below liver capsule and in a region without major vessels, bile ducts or masses	
Measurement in two different intercostal spaces	
Evenly distributed FOV colour	
Evenly spaced colour in ROI (no blue-red jumps)	
High quality measurement according to the manufacturer’s instrucitons^f^	
Diameter of perihepatic fluid between probe and liver < 20 mm	

^a^*IQR* interquartile range, ^b^*LSM* liver stiffnes measurement, ^c^*ROI* region of interest, ^d^*FOV* field of vision, ^e^*SD* standard deviation. ^f^see Fig. 1

Patients who did not have a reliable elastographic measurement were not included in further analyses. Data on the cause of failed or unreliable measurements were recorded. The final elastographic result (liver stiffness measurement - LSM) was provided in kilopascals (kPa) along with the simultaneously recorded IQR/LSM value (%), region of interest (ROI) diameter (mm), and average measurement depth (mm). The value of the measurement was not limited upward.

The morphological signs of cirrhosis were as follows: Uneven surface and/or rounded liver edge assessed by a linear monocrystalline probe of the same apparatus; left hepatic lobe or caudate lobe hypertrophy, and signs of fibrously altered parenchyma. US portosystemic collaterals visualization and splenomegaly were used as confirmative PH signs (Table 2). These signs were used only as an inclusion criterion and never as an exclusion criterion. In unclear cases regarding the presence of liver cirrhosis or a sinusoidal form of PH, a transjugular liver biopsy (TJLB) with concomitant measurement of portal pressure (HVPG) was performed.

**Table 2:** Ultrasound signs of cirrhosis and portal hypertension

Ultrasound signs of cirrhosis and portal hypertension	
Nodular liver surface	
Blunt liver edge	
Coarse echopattern	
Left lobe/right lobe ratio > 1.30	
Caudate lobe/right lobe ratio ≥ 0.65	
Reduction of the median segment of the left hepatic lobe	
Splenomegaly	
Visualisation of portosystemic collaterals	

### Statistical analysis

The value of the elastographic result was rounded to one decimal place, as was the IQR/LSM value. Internationally established units were used for laboratory data. Demographic and clinical population data, including laboratory data and 2D-SWE measurements, were recorded in an electronic database (SPSS Inc., Chicago, IL, USA) for statistical analysis. Descriptive summary statistics of the population were performed. Summary values were described as the average ± standard deviation (SD) and/or as a median, range, and interquartile range when appropriate (according to the distribution of the processed data). Comparisons between Groups 1 and 2 were performed using Spearman’s correlation method and using logistic regression (univariate and multivariate analyses). In order to evaluate the degree of LSM value separability, the probability of the LSM data in Groups 1 and 2 was evaluated using a receiver operating characteristic curve (ROC). The statistical analysis was conducted with SPSS 26.0 (SPSS Inc., Chicago, IL, USA). The alpha value was set to 0.05. All the *p*-values ​​were two-sided.

## Results

The main characteristics of the study population are provided in Table 3.

**Table 3:** Main study population’s characteristics

	Overall
	(*N* = 52)
**SEX**	
male	29 (56%)
female	23 (44%)
**Etiology**	
cirrhosis	34 (65%)
cancer	13 (25.0%)
other	5 (10%)
**Patient’s state**	
Dead	27 (52%)
Alive	25 (48%)
**SAAG > 11 g/L**	
yes	34 (65%)
No	18 (35%)
**Age (years)**	
Median (SD)	63 (12)
Min-Max	38–91
**ROI (mm)**	
Median (SD)	14.5 (1.3)
Min-Max	10–15
**Depth of the measurement (mm)**	
Median (SD)	42 (6.1)
Min-Max	30–50
**Trombocytes (per nL of blood)**	
Median (SD)	223 (174)
Min-Max	31–904
**ALT (IU/L)**	
Median (SD)	53 (38)
Min-Max	8–170
**Bilirubin (μmol/L)**	
Median (SD)	34 (29)
Min-Max	4–100
**INR**	
Median (SD)	1.4 (0.35)
Min-Max	0.9–2.3
**Serum albumin (g/L)**	
Median (SD)	30 (5)
Min-Max	19–43
**Ascites albumin (g/L)**	
Median (SD)	14 (8)
Min-Max	3–31
**SAAG**	
Median (SD)	16.3 (7.6)
Min-Max	4–32

^a^*SAAG* serum ascites albumin gradient, ^b^*SD* standard deviation, ^c^*INR* international normalized ratio, ^d^*mm* millimeters, ^e^*ROI* region of interest, ^f^*nL* nanoliter, ^g^*IU* international units

Our study included 34 patients (65%) with new-onset ascites with SAAG ≥11 g/L (Group 1). In these cases, cirrhosis and accompanying PH has been confirmed. Etiology of cirrhosis was due to harmful alcohol consumption (20 patients) and metabolically-induced steatohepatitis (8 patients) or a combination of both conditions. Cirrhosis was due to chronic HCV infection and primary biliary cholangitis in two patients.

Group 2 included 18 patients (35%) with new-onset ascites with SAAG < 11 g/L; ascites was due to cancer in 13 patients (25%) and in all cases with cytologically confirmed peritoneal carcinomatosis. Causes of peritoneal carcinomatosis included: upper and lower gastrointestinal cancer (four patients), ovarian cancer (three patients), pancreatic cancer (three patients), breast cancer (two patients), and primary peritoneal cancer (one patient). The remaining five patients (10%) had pancreatogenic ascites (two patients), tuberculosis (one patient), obscure cause (one patient), and immune-mediated ascites (one patient). To evaluate the presence of sinusoidal PH, three patients in Group 2 (5% of the total cohort) underwent an HVPG assessment. A total of 27 patients (52%) were deceased by the end of the study.

### SAAG and 2D-SWE relationship

**Fig. 2:**
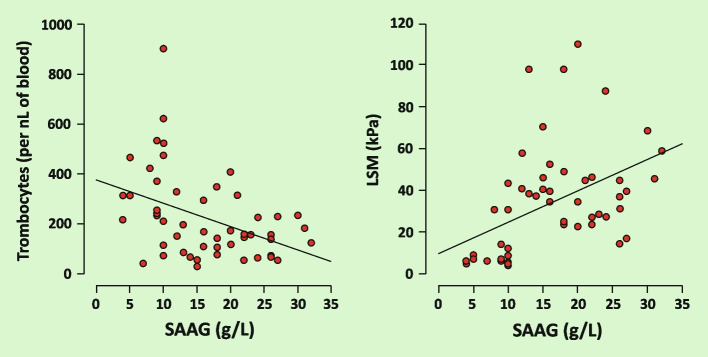
Relationship between trombocytes, LSM and SAAG according to Spearman’s analysis. LSM – liver stiffness measurement. SAAG – serum ascites albumin gradient. kPa – kilopascals. nL – nanoliter

Spearman’s analysis of the SAAG values correlation with the following variables was performed: LSM, depth of measurement, platelet count, ALT, and bilirubin. A positive association with LSM (rho = 0.55, 95% CI 0.31–0.72, *P* < 0.001), a negative association with platelet counts (rho = − 0.4, 95% CI -0.6 –0.13, *P* = 0.005) and a positive association with bilirubin level (rho = 0.52, 95% CI 0.28–0.69, P = *P* < 0.001) were demonstrated (Fig. 2).

SAAG ≥11 g/L showed a significant association with LSM (OR 1.15 (1.07–1.27); 95% confidence interval; *p* = 0.001). The LSM distribution in Group 1 was significantly higher than in Group 2 (*p* < 0.001, confirmed after adjustment for age and gender). Therefore, in the final analysis, we performed a ROC analysis predicting the LSM cut-off value when SAAG ≥11 g/L. The calculated cut-off value was 14.4 kPa (94% accuracy, 100% sensitivity, 83% specificity). In Group 1, the mentioned value defined all included patients (34 true positives, 0 false negatives). In Group 2, the mentioned value correctly defined 15 patients (15 true negatives, 3 false positives). The three false positive patients in Group 2 were patients who required invasive diagnostic evaluation (HVPG and transjugular liver biopsy) for a definitive diagnosis. The final diagnosis in these 3 cases were: extramedullary erythropoiesis in a patient with chronic myelogenous leukemia, porto-sinusoidal vascular disease (idiopathic PH) in 1 case, and ascites due to persistent leakage from the pancreatic duct injury in 1 case. The first two patients had SAAG < 11 g/L although we would expect it to be othervise. The clear reason behind the lower than expected SAAG value could not be recognized.

### Liver stiffness measurement by 2D-SWE

The distribution of liver stiffness measurement in the entire cohort in Groups 1 and 2 is provided in Table 4.

**Table 4:** Elastographic measurement parameters distribution

	Etiology		Overall
	Cirrhosis (*N* = 34)	Other (*N* = 18)	(*N* = 52)
**LSM** (kPa)			
Median (SD)	45.8 (23.6)	11.9 (11.2)	34.1 (25.8)
Min-Max	14.4–110	4.10–18.4	4.10–110
**IQR** (kPa)			
Median (SD)	9.62 (7.48)	1.83 (1.31)	6.93 (7.13)
Min-Max	1.20–35.0	0.400–5.60	0.400–35.0
**IQR/LSM** (%)			
Median (SD)	20.7 (7.66)	18.9 (5.95)	20.1 (7.11)
Min-Max	5.00–30.0	9.00–29.0	5.00–30.0

^a^*LSM* liver stiffness measurement, ^b^*IQR* interquartile range, ^c^*SD* standard deviation, ^d^*kPa* kilopascals

Measurement was attempted in 62 patients, and reliable elastographic measurement was performed in 52 patients (84% reliable measurement success rate). The main reasons for failed measurement were (ranked from most common to rare): obesity, depth of measurement, patients ability to hold their breath, insufficient liver volume, the influence of heart pulsatility on liver movement in ascites, insufficient ROI, and excessive end-result heterogeneity. The ultrasound machine’s inability to perform the measurement was observed in < 1% of all measurements. The reasons for the latter were not clearly identified.

## Discussion

The main finding of this study at a single secondary hospital center is that 2D-SWE of the liver can noninvasively discriminate between portal hypertension-related newly onset ascites. The proposed 14 kPa cut-off value is comparable to the values proposed for cirrhosis confirmation in the cACLD group [10, 18–20] and roughly comparable to the proposed values for noninvasive CSPH exclusion criteria in published studies and meta-analyses (cACLD populations) [6, 10–15, 24]. The result has important clinical value since our study defines liver elastography (noninvasive, bedside, widely available method), especially its 2D-SWE version, as the best investigative method for assesing CSPH in patients with newly formed ascites. This definition applies mainly to patients with dACLD in the stable disease phase and may be important for further study in patients with severe CSPH (HVPG > 16 mmHg). According to our analysis, the close relationship between 2D-SWE and CSPH is also indirectly indicated by the characteristic association between SAAG, 2D-SWE, and platelet blood count. Thrombocytopenia is one of the characteristic CSPH consequences and is involved in most of the proposed noninvasive CSPH assessments guidelines [10].

Similar conclusions were reached by Bota et al. in a larger sample of patients where the liver fibrosis assessment was performed using pSWE [17]. Due to a different design (strictly sequential patients inclusion), their study included significantly more patients with cirrhosis and a comparable number of patients in the exudative ascites group. The cut-off values ​​of their study and ours differ (approximately 10 kPa in Botaet al. versus 14 kPa in our cohort). An important reason for this difference may be the used elastographic method, as 2D measurement performs liver stiffness analysis on a much larger liver parenchyma sample. Furthermore, the Bota et al. cohort does not clearly state whether the cut-off value analysis was performed under the SAAG < or ≥ 11 g/L condition. Furthermore, patients with cirrhosis and the rest of the group ratios are significantly different (approximately 3:1 in Bota et al. versus 2:1 in our study). The accuracy of the proposed cut-off value between their cohort and ours was otherwise comparable (95% accuracy versus 94%).

Our study’s calculated 14 kPa cut-off value had an appropriate exclusion value for identifying patients with ascites not due to PH, especially in the patient group with peritoneal carcinomatosis resulting from metastatic cancer. Together with some other study data in this area [25–34] – elastography can provide additional non invasive diagnostic information.

Although the analysis was performed in a minimal number of patients, the 2D-SWE exclusion value determining the cause of ascites in groups other than liver cirrhosis or peritoneal carcinomatosis appears to be less accurate, especially in cases where etiologic differentiation was difficult and required extensive and invasive diagnostic work-up. A study by Bota et al. met similar findings [17]. Furthermore, 2D-SWE does not provide sufficient information on mixed ascites, where paracentesis is always required for a definitive determination. The same applies to the cytological pattern analysis and inflammatory process evaluation in ascites [35–41].

Our study used SAAG, a commonly used sum in this field, to divide the ascites cause. A study on several patients by Li et al. used a serum ascites cholesterol gradient (SACG) to determine the cause of ascites. SACG proved to be comparable to SAAG in the PH ascites group and more sensitive in differentiating the cause in the mixed ascites and non-PH ascites group but was not evaluated in our study [42].

Regarding the 2D-SWE measurement using MyLab Nine and its clinical applicability, our study shows about an 85% success rate in performing reliable measurements. This percentage is significantly lower than those reported in studies that evaluated liver cACLD cohorts (typically > 90 or even 95% applicability) [14–16, 24, 43, 44]. There are several reasons for this finding. In our study, the elastographic measurement was performed exclusively in patients with ascites. According to some studies, ascites should not significantly affect the elastographic result quality [14–16, 24, 44]. On the other hand, according to Zhang et al., it had a significant effect on both reliability and measurement quality, showing a similar effect on the end result as ROI size [45], with which the authors of our study agree.

It is also important to mention the lack of a protocol for reliable 2D-SWE [22]. The protocol in our study was a sum of study recommendations and past findings in this area [18–22]. The ultrasound machine type (MyLab Nine) also had a significant impact because there is no official validation yet compared with commonly available devices on the market (Supersonic Imagine, France) [20]. The results between different measurement systems can vary between 6 and 12% [19]. A recently published study provided head to head 2D-SWE comparison between MyLab Nine and Supersonic Imagine in a cACLD population in order to assess liver fibrosis staging tresholds with very good proven correlation [46]. To our knowledge, our study is the first one to evaluate the MyLab Nine 2D-SWE measurement in the dACLD group. The most important causes of unreliable measurement were comparable to those defined to date; however, the influence of the measurements’ depth, especially with concomitant overweight, bilirubin level, patient participation, and the heart function’s influence on liver movement during each measurement, is more pronounced in patients with significant ascites [15, 18, 19, 21, 47–50].

Our study has important limitations, which we must consider when interpreting the results. Our study was conducted in one hospital center with a relatively small group of patients and retrospective data analysis. Given these findings, 2D shear wave liver elastography has potential in evaluating new-onset ascites but needs further validation in a larger prospective study. Patients in our cohort had strictly defined inclusion and exclusion criteria due to the potential impact on the SAAG and 2D-SWE results. In our estimation, this decision may have excluded up to 30% of the total patients with newly onset ascites. Elastographic measurement was also performed by a single investigator and on an US apparatus that is not study validated, meaning that we do not have data on inter-observer reproducibility and on head to head comparison with the validated US soft-ware. In the group of patients with transudation resulting from PH and cirrhosis, we did not precisely define the liver cirrhosis etiology influence on the ascites formation and elastographic measurement. Recent data suggest that, especially in the group with metabolically-induced liver cirrhosis, ascites in a small group of patients develop in parallel or before the proposed HVPG threshold values [50]. Also, it is important to note, that our study does not provide direct 2D-SWE comparison between dACLD and patients with normal liver stiffness which can explain why no false negatives were detected when performing ROC analysis in the Group 1. As such, it can not be fully translated to the populations with low PH-related ascites prevalence. The presence of free fluid is likely to make the accuracy of elastography suboptimal. Since there is no comparative clinical study and no gold standard to demonstrate the elastography accuracy in patients with ascites, we have to carefully interpret the results even in the most reliable measurements cases. Finally, paracentesis is still a required diagnostic procedure when evaluating new-onset ascites and can probably paradoxically enhance 2D-SWE quality as already mentioned.

## Conclusions

The results of our study underline the feasibility, and good applicability of a simple and readily available ultrasound method to assess the PH-related cause of newly onset ascites at the bedside. Also, 2D-SWE held the accuracy necessary for differentiating between transudative ascites in patients with PH related to liver cirrhosis and exudative ascites in patients with underlying peritoneal carcinomatosis. In our view, the routine use of this method during ultrasound examinations of patients with newly onset ascites should be attempted to enable quick, noninvasive identification.

## Data Availability

Manuscript data and materials are available from the corresponding author on reasonable request.

## Abbreviations

PH: Portal hypertension
SAAG: Serum ascites albumin gradient
CSPH: Clinically significant portal hypertension
cACLD: compensated advanced chronic liver disease
TE: Transient elastography
2D-SWE: 2D shear wave elastography
pSWE: Point shear wave elastography
US: Ultrasound
CT: Computed tomography
AST: Aspartate aminotransferase
ALT: Alanine aminotransferase
ACLF: Acute on chronic liver failure
LSM: Liver stiffness measurement
IQR: Interquartile range
ROI: Region of interest
TJLB: Transjugular liver biopsy
HVPG: Hepatic vein pressure gradient
SD: Standard deviation
ROC: Receiver operating characteristic
dACLD: Decompensated advanced chronic liver disease
MRI: Magnetic resonance imaging
CRP: C-reactive protein
TNF-alpha: Tumor necrosis factor alpha
CEA: Carcinoembryonic antigen
IGF: Insulin-like growth factor.
VEGF: Vascular endothelial growth factor
SACG: serum ascites cholesterol gradient
